# *In Vitro* Activity of Omadacycline and Five Comparators against Contemporary Ribotypes of Clostridioides difficile in Stockholm, Sweden

**DOI:** 10.1128/Spectrum.01440-21

**Published:** 2021-10-06

**Authors:** Angela Camporeale, Chaitanya Tellapragada, Jelena Kornijenko, Carl Erik Nord, Christian G. Giske

**Affiliations:** a Department of Laboratory Medicine, Division of Clinical Microbiology, Karolinska Institute t, Stockholm, Sweden; b Department of Clinical Microbiology, Karolinska University Hospital, Stockholm, Sweden; University of Texas Southwestern Medical Center

**Keywords:** agar dilution method, *Clostridioides difficile*, omadacycline

## Abstract

Clostridioides difficile infection represents a growing clinical challenge. The new compound omadacycline is a potential treatment alternative, as many antibiotics have limited activity or are rarely used due to costs and side effects. The activity of omadacycline and five comparators was assessed with agar dilution on a 2015-to-2018 collection of 65 C. difficile isolates from Sweden. Omadacycline demonstrated *in vitro* activity against the contemporary ribotypes of C. difficile, and further clinical investigation is needed.

**IMPORTANCE** Evaluating the activity of novel antimicrobials like omadacycline is of great interest, as a reliable and efficient antimicrobial treatment for Clostridioides difficile infections is in demand.

## OBSERVATION

Clostridioides difficile is an important nosocomial pathogen worldwide, causing C. difficile infection (CDI). Historically, metronidazole, given orally, has been the mainstay therapeutic agent for the treatment of CDI. A low efficacy of metronidazole among patients with a severe form of the disease and a high recurrence rate among patients despite receiving adequate treatment has prompted the need for treatment alternatives. Even though recurrence rates remain similar, comparative studies proved that vancomycin was superior, and therefore, it is now preferred as an initial treatment (1, 2). Additionally, fidaxomicin has been approved for CDI treatment and tigecycline was evaluated for treatment of patients with CDI. However, fidaxomicin is costly and does not seem to have a window for amortization due to its narrow use, whereas tigecycline is known for its side effects; thus, both are restricted in their utility (3, 4). Omadacycline is an aminomethylcycline antibiotic belonging to the tetracycline class that has been synthesized by chemical modifications of minocycline in order to overcome the most common class-related drug resistance mechanisms (efflux and ribosomal protection) found in bacterial pathogens (5). Several studies have previously reported that omadacycline has potent *in vitro* activity against a range of aerobic bacteria and a few anaerobic bacterial species (6–8). After FDA approval for the treatment of patients with acute bacterial skin and skin structure infections and community-acquired bacterial pneumonia in 2018, the clinical efficacy and safety of omadacycline were recently reported (9). Furthermore, omadacycline was reported as a potential therapeutic agent for CDI due to its ability to not induce toxin production and proliferation of C. difficile in an *in vitro* gut model (10). Herein, we report the *in vitro* activity of omadacycline and five comparators against a contemporary collection of C. difficile isolates from Stockholm, Sweden.

(Preliminary results were submitted as an abstract for ECCMID 2020.)

A collection of clinical C. difficile isolates obtained from patients diagnosed with CDI in the Stockholm County region between 2015 and 2018 was used in the present study. Data regarding the contemporary and prevalent ribotypes (RTs) in Sweden during 2015 to 2018 were extracted from the Swedish Public Health Agency database. C. difficile isolates belonging to nine different ribotypes were obtained from the Clinical Microbiology Laboratory, Karolinska University Hospital, Huddinge, Stockholm, Sweden, and one isolate belonging to ribotype 027 was obtained from the Department of Clinical Microbiology, Jönköping Hospital, Jönköping, Sweden. A reference strain of C. difficile, ATCC 43594 (ribotype 005), was used for quality control. All isolates were freshly subcultured before testing and confirmed as C. difficile using matrix-assisted laser desorption ionization–time of flight mass spectrometry (MALDI-TOF MS) (Bruker Diagnostics). The antibiotics tested alongside omadacycline were metronidazole, vancomycin, fidaxomicin, tigecycline, and moxifloxacin; these were incorporated within blood agar at various concentrations (Table 1) to perform the agar dilution method. Briefly, bacterial suspensions at 0.5 McFarland were grown on the aforementioned plates at 37°C for 48 h under anaerobic conditions. The MICs for omadacycline and comparator agents were evaluated according to the breakpoints and/or the epidemiological cut-offs (ECOFFs) recommended by EUCAST (https://www.eucast.org/fileadmin/src/media/PDFs/EUCAST_files/Breakpoint_tables/v_11.0_Breakpoint_Tables.pdf, accessed 4 January 2021). The values for all the study isolates were determined in triplicate, and the geometric mean MICs, MIC_50_s, and MIC_90s_ are reported.

**TABLE 1:** Susceptibility overview of 65 C. difficile isolates for 9 antibiotics

Antimicrobials	Breakpoint (mg/liter)^*a*^	No. (%) of isolates with MIC (mg/liter) of:													MIC_50_^*b*^	MIC_90_^*b*^	GM MIC^*c*^
		≤0.008	0.015	0.03	0.06	0.12	0.25	0.5	1	2	4	8	16	32			
Omadacycline	—	0 (0)	0 (0)	0 (0)	0 (0)	0 (0)	3 (4.6)	41 (63.1)	17 (26.1)	0 (0)	2 (3.1)	1 (1.5)	1 (1.5)	NT	0.25/0.5	1	0.67
Metronidazole	≥2	NT^*d*^	NT	0 (0)	0 (0)	1 (1.5)	4 (6.1)	49 (75.3)	8 (12.3)	2 (3.1)	1 (1.5)	NT	NT	NT	0.25/0.5	1	0.55
Vancomycin	≥2	NT	NT	NT	NT	NT	0 (0)	0 (0)	55 (84.6)	6 (9.2)	4 (6.1)	NT	NT	NT	0.5/1	2	1.16
Fidaxomicin	—	0 (0)	0 (0)	2 (3.1)	11 (16.9)	18 (27.6)	24 (36.9)	8 (12.3)	0 (0)	2 (3.07)	NT	NT	NT	NT	0.12/0.25	0.5	0.17
Tigecycline	≥0.25	NT	NT	NT	55 (84.6)	7 (10.7)	1 (1.5)	1 (1.5)	0 (0)	1 (1.5)	0 (0)	NT	NT	NT	0.03/0.06	0.12	0.07
Moxifloxacin	≥4	NT	NT	NT	NT	NT	NT	0 (0)	0 (0)	35 (53.8)	23 (35.3)	7 (10.7)	NT	NT	2	4/8	2.99

a ECOFFs established by EUCAST were used where available. —, no breakpoint established.

b A range of concentrations is given for ambiguous MIC_50_ and MIC_90_ interpretation.

c GM, geometric mean.

d NT, concentration not tested.

Sixty-five clinical C. difficile isolates were tested. The ribotypes included were 014 (*n* = 20 isolates), 002 (*n* = 11), 023 (*n* = 8), 001 (*n* = 7), 005 (*n* = 5), 078 (*n* = 5), 020 (*n* = 4), 078/126 (*n* = 3), 027 (*n* = 1), and 012 (*n* = 1). The distribution of the study isolates based on their MICs for the antibiotics tested is depicted in (Table 1). The geometric mean MIC, MIC_50_, and MIC_90_ for omadacycline were 0.677 mg/liter, 0.5 mg/liter, and 1 mg/liter respectively. The MIC_50_ and MIC_90_ of omadacycline reported from the present study are comparable with the results (MIC_50_, 0.25 mg/liter, and MIC_90_, 0.5 mg/liter) from a previous study that used the agar dilution method for MIC determination among 21 clinical isolates of C. difficile (8). In contrast, our results are higher than the MIC_50_ (0.031 μg/ml) and MIC_90_ (0.031 μg/ml) reported in a more recent study from Texas, USA (11). The ribotypes tested and, more importantly, the method (broth microdilution) employed for MIC determination in the study reported by Begum et al. were different from the ribotypes and method in our study. Moreover, broth microdilution has been proven to show discrepancies for anaerobic bacteria, especially C. difficile; hence, the results from the two studies should be compared with caution (12).

Among the five comparators tested in the present study, four antimicrobials have established ECOFFs and none of them have clinical breakpoints reported by EUCAST. Given this context, we did not use the standard susceptible-intermediate-resistant (S-I-R) definitions for characterizing the study isolates against the individual antibiotics tested. The geometric mean MIC of omadacycline (0.67 mg/liter) was comparable with the geometric mean MIC of metronidazole (0.55 mg/liter) and higher than the values for fidaxomicin (0.17 mg/liter) and tigecycline (0.07 mg/liter) among our strain collection. Despite the higher geometric mean MICs for omadacycline in comparison with those of fidaxomicin and tigecycline, the new agent can be a potential candidate for treatment of CDI due to its favorable pharmacokinetic and pharmacodynamic properties and its limited impact on the normal gut microbiota (10, 13, 14). Substantial variations in the geometric mean MICs were not observed among different ribotypes, with values ranging within 2 or 3 absolute 2-fold concentrations for all the antibiotics tested (Table 2). However, the geometric mean MICs for omadacycline were slightly higher among strains of ribotypes 078/126, 001, and 005 than among the other ribotypes tested in the present study. Furthermore, one isolate each belonging to ribotypes 078/126 and 014 had omadacycline MICs of 8 and 16 mg/liter, respectively. We foresee the need for further genetic characterization of these isolates to study the underlying mechanisms for higher MICs of omadacycline in these two isolates. One limitation of the present study is that we could determine the MIC of omadacycline against only one isolate of C. difficile belonging to the well-established virulent ribotype 027, which is more prevalent elsewhere. Infections caused by C. difficile ribotype 027 are still not common in the Stockholm region (15), and hence, these strains were unavailable for testing. In summary, omadacycline demonstrated *in vitro* activity against a contemporary ribotype collection of C. difficile isolates from Sweden. Our results are promising but suggest the need for susceptibility testing of a larger and more internationally diverse group of C. difficile strains and further investigation of the mechanism of high MICs observed in selected isolates.

**TABLE 2:** Geometric mean MIC by ribotype

Ribotype or strain (no. of isolates tested)	Geometric mean MIC (mg/liter) (unless otherwise indicated) for:					
	Omadacycline	Metronidazole	Vancomycin	Fidaxomicin	Tigecycline	Moxifloxacin
Total (65)	0.67	0.55	1.16	0.17	0.07	2.99
001 (7)	0.84	0.82	1.48	0.13	0.1	2.60
002 (11)	0.53	0.53	1.21	0.28	0.06	3.24
005 (5)	0.72	0.52	1.44	0.18	0.06	2.76
012 (1)	0.5	0.5	1.58	0.08	0.06	8
014 (20)	0.64	0.53	1.12	0.12	0.07	3.4
020 (4)	0.59	0.74	1.18	0.19	0.06	4.23
023 (8)	0.45	0.35	1.02	0.11	0.06	2.51
027 (1)	0.5	0.5	1	0.5	0.06	2
078 (5)	0.58	0.95	1.66	0.19	0.09	2.40
078/126 (3)	1.31	0.62	1.25	0.24	0.09	4.32
ATCC 43594^*a*^	1	0.5	2	0.25	0.06	2

a MICs are given instead of geometric mean MICs for strain ATCC 43594.
